# Association between pesticide use and liver injury: A field study in Taiwan

**DOI:** 10.5271/sjweh.4311

**Published:** 2026-09-01

**Authors:** Yu-Chang Liu, Tsung-Cheng Kuo, How-Ran Guo

**Affiliations:** 1 Department of Emergency Medicine, Chi Mei Medical Center, Tainan, Taiwan.; 2 Department of Senior Welfare and Services, College of Smart Health, Southern Taiwan University of Science and Technology, Tainan, Taiwan.; 3 Department of Environmental and Occupational Health, College of Medicine, National Cheng Kung University, Tainan, Taiwan.; 4 Department of Occupational and Environmental Medicine, National Cheng Kung University Hospital, Tainan, Taiwan.

**Keywords:** agricultural worker, hepatitis, liver injury, occupational exposure, pesticide

## Abstract

**Objectives:**

Pesticides are crucial for agricultural development but can be harmful to humans. We conducted a study to investigate the association between pesticide use and liver injury.

**Methods:**

We recruited participants ≥40 years from a district with two agricultural regions where water caltrops and mangoes are cultivated. A random sample of 200 residents from each region was selected, and prevalence ratios (PR) for abnormal liver tests were compared.

**Results:**

A total of 331 individuals participated (participation rate: 82.8%), with 151 from the water caltrop region and 180 from the mango region. Most participants were ≥60 years and predominantly female. Pesticide users had a higher risk of abnormal liver tests [PR 1.74, 95% confidence interval (CI) 1.15–2.62]. Stratified analyses showed higher risks among those with a positive HBsAg test (PR 2.50, 95% CI 1.34–4.64) or a positive anti-HCV Ab test (PR 10.61, 95% CI 6.00–18.77). Multivariable analysis identified a positive HBsAg test [adjusted PR (PR_adj_) 3.21, 95% CI 1.38–7.48], a positive anti-HCV Ab test (PR_adj_ 23.52, 95% CI 11.30–48.96), and upward pesticide spraying (PR_adj_ 3.39, 95% CI 1.52–7.54) as independent risk factors for abnormal liver tests.

**Conclusions:**

Pesticide use was associated with a higher risk of liver injury, particularly among upward sprayers and those with hepatitis B or C infection. The proportion of abnormal liver test cases attributable to upward pesticide spraying in this population might be as large as hepatitis B, supporting the importance of pesticide exposure.

Pesticides play a crucial role in modern agriculture by improving crop yields and food security in a cost-effective manner, minimizing crop losses and increasing the availability of high-quality food products (1). However, because pesticides are inherently toxic by design, unintended exposure to these compounds poses significant health risks to humans and other organisms (2). Humans may be exposed directly during occupational activities or indirectly through contaminated air, water, soil, and food (3). The primary routes of pesticide entry into the human body include dermal absorption, ocular contact, oral ingestion, and inhalation (4). Workers in pesticide-related industries and agriculture face higher exposure risks compared to the general population (3).

Pesticide exposure can cause both acute toxic effects and long-term health consequences (5). These chemicals have been linked to various forms of toxicity – including carcinogenic, neurotoxic, reproductive, metabolic, pulmonary, and developmental effects – resulting in a wide spectrum of chronic diseases (6). The liver is a vital organ responsible for a wide range of physiological functions, including immunity, digestion, detoxification, and metabolic regulation (7). During phase I microsomal oxidation, many pesticides undergo bioactivation rather than detoxification, resulting in the generation of reactive toxic metabolites that can cause hepatic injury (8, 9). When hepatocytes are damaged, increased membrane permeability or cell necrosis leads to the release of alanine aminotransferase (ALT) and aspartate aminotransferase (AST) into the bloodstream (10). These liver enzymes serve as valuable biomarkers for detecting and monitoring liver injury.

Animal studies have demonstrated that pesticide exposure alters liver enzyme levels (11, 12). In humans, some studies have found elevated ALT and AST levels among workers with long-term occupational pesticide exposure compared to unexposed controls (13–15). However, other studies have reported decreased or unchanged enzyme levels in pesticide users (16, 17). These conflicting findings highlight the need for further investigation into the relationship between pesticide use and abnormal liver enzymes. The present study aimed to investigate the association between pesticide use and liver injury in an agricultural population in Taiwan. In addition to pesticide exposure itself, we also evaluated potential contributing factors including viral hepatitis infection and pesticide spraying patterns.

## Methods

### Study setting and population

**Figure 1:**
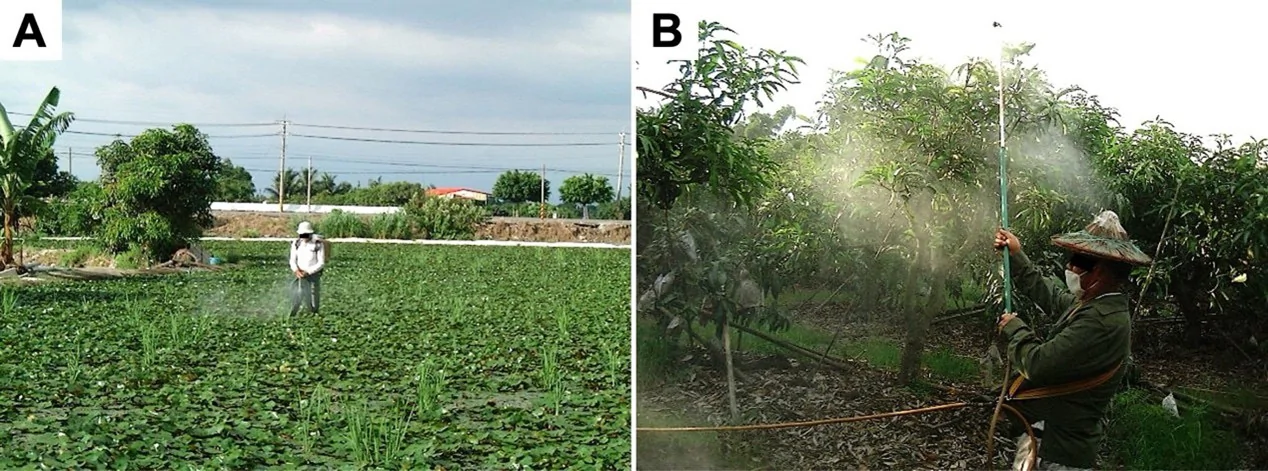
Pesticide spraying methods employed in the two agricultural regions. (A) Downward spraying method used by water caltrop farmers, with the spray gun directed toward the ground. (B) Upward spraying method used by mango farmers, with the spray gun directed toward the tree canopy.

This study was conducted in the Guantian District of Tainan City, located in southern Taiwan. Guantian is a rural area known for cultivating water caltrops and mangoes. Although both crops require frequent pesticide application, the spraying methods differ substantially. Because water caltrops are cultivated in ponds, farmers typically spray pesticides downward by directing the spray gun toward the ground (figure 1A). In contrast, mangoes are grown on trees, so farmers generally spray pesticides upward by directing the spray gun toward the tree canopy (figure1B). This upward spraying pattern causes pesticides to pass through the applicator’s breathing zone, resulting in substantially higher potential inhalation exposure.

We identified all individuals aged ≥40 years from the list of household registries maintained by the Guantian District Household Registration Office. These individuals were assigned to one of two groups according to residential location: the water caltrop growing region (N=1168) and the mango growing region (N=2168). Residents of these two agricultural regions had similar lifestyles and work patterns. A random sample of 200 individuals was drawn from each region. All sampled individuals were invited to participate in this study during a regular health examination provided under Taiwan’s Health Promotion Administration program for adults aged ≥40 years. The Guantian District Health Center conducted the health examinations, and the Health Promotion Administration covered the costs without additional charge. The study protocol complied with the principles of the Declaration of Helsinki and was approved by the Research Proposal Review Committee of the Department of Environmental and Occupational Health, National Cheng Kung University (no. S76861103). The study objectives, methods, and potential risks were explained to the participants in the local language, and written informed consent was obtained from each participant.

### Data collection

A structured questionnaire was administered to collect personal information, including age, sex, alcohol consumption, smoking status, comorbidities, pesticide use, and pesticide spraying patterns. Participants were categorized into four age groups: 40–49, 50–59, 60–69, and ≥70 years. Alcohol consumption was classified into five categories: non-drinker (never consumed alcoholic beverages), former infrequent drinker (<2 drinks per week previously but abstained during the year before the examination), former regular drinker (≥2 drinks per week previously but abstained during the year before the examination), current infrequent drinker (currently consumes <2 drinks per week), and current regular drinker (currently consumes ≥2 drinks per week). Smoking status was classified into three categories according to widely used epidemiological definitions (18): non-smoker (smoked <100 cigarettes in their lifetime), former smoker (smoked ≥100 cigarettes in their lifetime but not currently smoking), and current smoker (smoked ≥100 cigarettes in their lifetime and currently smoking). Pesticide spraying patterns were classified as non-sprayer, downward spraying, and upward spraying.

Fasting blood samples were collected from each participant and analyzed at a certified clinical laboratory for ALT, AST, hepatitis B surface antigen (HBsAg), and anti-hepatitis C virus antibody (anti-HCV Ab). The upper limits of normal for both AST and ALT were defined as 40 U/L based on laboratory reference ranges. A positive HBsAg result indicated current hepatitis B virus infection, while a positive anti-HCV Ab result indicated past or present hepatitis C virus infection (19, 20).

### Outcome measures

The primary outcome was liver injury, defined as elevation of either AST or ALT above the upper limit of normal (20). Participants meeting this criterion were classified as having abnormal liver tests. The prevalence of abnormal liver tests was calculated and used as the outcome measure for overall and subgroup analyses.

### Statistical analysis

Categorical variables of demographic characteristics were summarized as counts and percentages for each residential region, and differences between groups were assessed using Pearson’s chi-square test. The prevalence of abnormal liver tests was calculated for each exposure group, and prevalence ratios (PR) with 95% confidence intervals (CI) were estimated as measures of association. Stratified analyses by pesticide use and viral hepatitis status were performed to examine potential effect modification. Multivariable log-binomial regression models were employed to identify independent predictors of abnormal liver tests. To assess the robustness of our findings, sensitivity analyses were performed. The E-value was calculated to quantify the minimum strength of association that an unmeasured confounder would need to have with both the exposure and outcome to fully explain away the observed association (21). To estimate the potential population-level impact under the assumption of a causal relationship, the population attributable fractions (PAF) were calculated using Levin’s formula (22). A two-tailed P-value of <0.05 was considered statistically significant. Statistical analyses were performed using Statistical Analysis System (SAS) 9.4 for Windows (SAS Institute, Inc., Cary, NC, USA).

## Results

### Characteristics of study population

**Figure 2:**
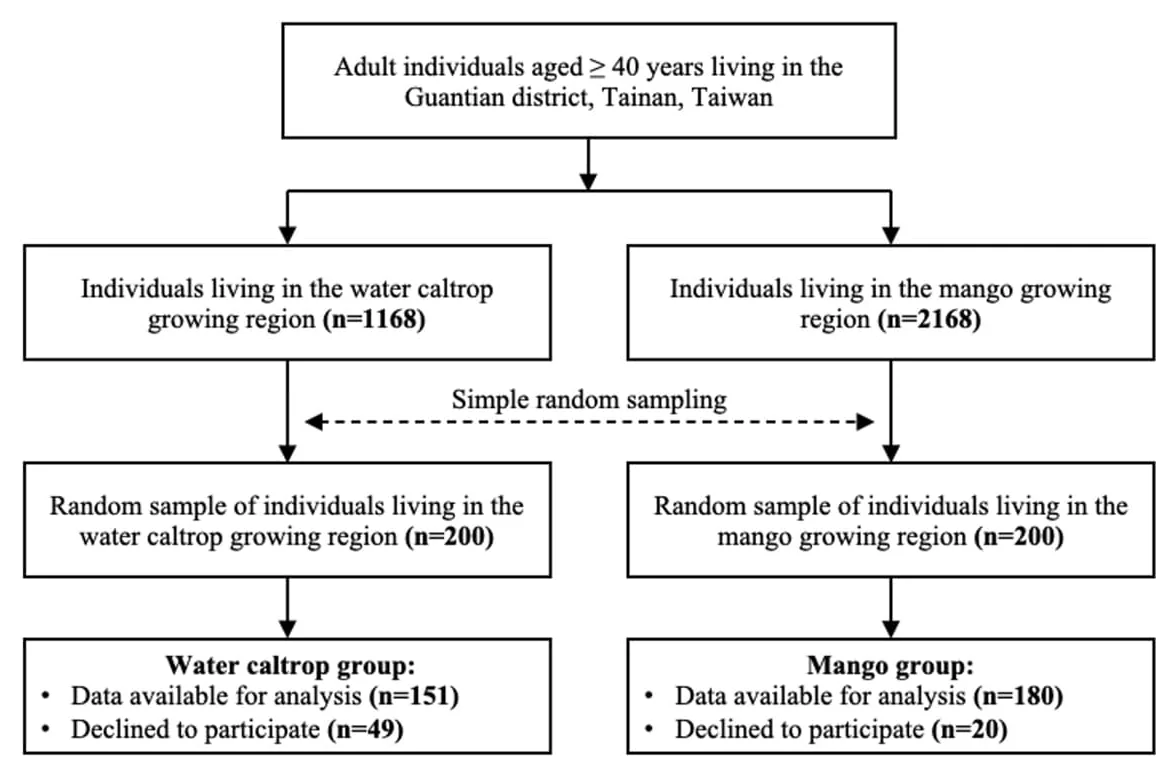
Flow diagram of participant recruitment and inclusion in the study.

Among the 400 adults sampled, a total of 331 (82.8%) participated in this study (figure 2). There were 151 participants in the water caltrop region group and 180 participants in the mango region group (table 1). The majority of participants were in the age subgroups of ≥60 years (70.2% and 65.0% in each group, respectively) with a predominance of female participants (53.6% and 53.3% in each group, respectively). The distributions of sex, age, alcohol consumption, smoking status, and pesticide use were comparable between the two groups. However, differences in the prevalence of abnormal liver tests, pesticide spraying patterns, and prevalence of hepatitis C infection reached statistical significance (P<0.05 for all). Participants in the mango growing region had a higher prevalence of abnormal liver tests (27.8% versus 15.2%, P=0.006) and hepatitis C infection (23.9% versus 10.6%, P=0.002). The two regions exhibited distinct pesticide spraying patterns (P<0.001): nearly all pesticide users in the water caltrop region employed the downward spraying method (96.6%), whereas those in the mango region predominantly used the upward spraying method (68.6%).

**Table 1:** Comparison of demographic characteristics between participants from water caltrop and mango growing regions. [HBsAg=hepatitis B surface antigen; Anti-HCV Ab=anti-hepatitis C virus antibody; AST=aspartate aminotransferase; ALT=alanine aminotransferase].

		Water caltrop region (N=151)		Mango region (N=180)	P-value
		N (%)		N (%)	
Sex					0.955
	Female	81 (53.6)		96 (53.3)	
	Male	70 (46.4)		84 (46.7)	
Age (years)					0.766
	40–49	15 (9.9)		22 (12.2)	
	50–59	30 (19.9)		41 (22.8)	
	60–69	63 (41.7)		67 (37.2)	
	≥70	43 (28.5)		50 (27.8)	
Alcohol consumption					0.210
	Non-drinker	108 (71.5)		127 (70.6)	
	Former infrequent drinker	6 (4.0)		18 (10.0)	
	Former regular drinker	7 (4.6)		8 (4.4)	
	Current infrequent drinker	17 (11.2)		18 (10.0)	
	Current regular drinker	13 (8.6)		9 (5.0)	
Smoking status					0.325
	Non-smoker	109 (72.2)		124 (68.9)	
	Former smoker	11 (7.3)		22 (12.2)	
	Current smoker	31 (20.5)		34 (18.9)	
Pesticide use					0.112
	Non-user	92 (60.9)		94 (52.2)	
	User	59 (39.1)		86 (47.8)	
Pesticide spraying pattern					<0.001
	Non-sprayer	92 (60.9)		94 (52.2)	
	Downward spraying ^†^	57 (37.8)		27 (15.0)	
	Upward spraying ^‡^	2 (1.3)		59 (32.8)	
Liver tests ^§^					0.006
	Normal	128 (84.8)		130 (72.2)	
	Abnormal	23 (15.2)		50 (27.8)	
HBsAg					0.236
	Negative	135 (89.4)		153 (85.0)	
	Positive	16 (10.6)		27 (15.0)	
Anti-HCV Ab					0.002
	Negative	135 (89.4)		137 (76.1)	
	Positive	16 (10.6)		43 (23.9)	

^†^ Downward spraying: pesticide application with spray gun directed toward the ground. ^‡^ Upward spraying: pesticide application with spray gun directed toward the tree canopy. ^§^ Abnormal liver tests defined as AST or ALT >40 U/L.

### Risk factors for abnormal liver tests

Our analysis revealed that the risk of abnormal liver tests was not associated with sex, age, alcohol consumption, or smoking status (table 2). There were associations of abnormal liver tests with pesticide use [PR 1.74; 95% confidence interval (CI) 1.15–2.62), upward spraying of pesticides (PR 2.36, 95% CI 1.50–3.69), and hepatitis C infection (PR 6.25, 95% CI 4.32–9.03). Participants with hepatitis B infection tended to have higher prevalence of abnormal liver tests (PR 1.59, 95% CI 0.98–2.59), with the increase reaching borderline statistical significance (P=0.062).

**Table 2:** Prevalence and risk of abnormal liver tests by participant characteristics. [CI=confidence interval; HBsAg=hepatitis B surface antigen; Anti-HCV Ab=anti-hepatitis C virus antibody; AST=aspartate aminotransferase; ALT=alanine aminotransferase].

		Abnormal liver tests^†^ N (%)	Prevalence ratio (95% CI)	P- value
Sex				
	Female	35 (19.8)	1.00 (Reference)	–
	Male	38 (24.7)	1.25 (0.83–1.87)	0.284
Age (years)				
	40–49	8 (21.6)	1.00 (Reference)	–
	50–59	20 (28.2)	1.31 (0.64–2.67)	0.470
	60–69	31 (23.9)	1.11 (0.56–2.19)	0.780
	≥70	14 (15.1)	0.70 (0.32–1.52)	0.363
Alcohol consumption				
	Non-drinker	54 (23.0)	1.00 (Reference)	–
	Former infrequent drinker	5 (20.8)	0.91 (0.40–2.05)	0.813
	Former regular drinker	1 (6.7)	0.29 (0.04–1.96)	0.204
	Current infrequent drinker	6 (17.1)	0.75 (0.35–1.60)	0.453
	Current regular drinker	7 (31.8)	1.38 (0.72–2.67)	0.330
Smoking status				
	Non-smoker	53 (22.8)	1.00 (Reference)	–
	Former smoker	6 (18.2)	0.80 (0.37–1.71)	0.564
	Current smoker	14 (21.5)	0.95 (0.56–1.59)	0.837
Agricultural region				
	Water caltrop region	23 (15.2)	1.00 (Reference)	–
	Mango region	50 (27.8)	1.82 (1.17–2.84)	0.008
Pesticide use				
	Non-user	31 (16.7)	1.00 (Reference)	–
	User	42 (29.0)	1.74 (1.15–2.62)	0.008
Pesticide spraying pattern				
	Non-sprayer	31 (16.7)	1.00 (Reference)	–
	Downward spraying ^‡^	18 (21.4)	1.29 (0.76–2.16)	0.344
	Upward spraying ^§^	24 (39.3)	2.36 (1.50–3.69)	<0.001
HBsAg				
	Negative	59 (20.5)	1.00 (Reference)	–
	Positive	14 (32.6)	1.59 (0.98–2.59)	0.062
Anti-HCV Ab				
	Negative	31 (11.4)	1.00 (Reference)	–
	Positive	42 (71.2)	6.25 (4.32–9.03)	<0.001

^†^ Abnormal liver tests defined as AST or ALT >40 U/L. ^‡^ Downward spraying: pesticide application with spray gun directed toward the ground. ^§^ Upward spraying: pesticide application with spray gun directed toward the tree canopy.

Stratified analyses revealed that pesticide use was associated with abnormal liver tests in both participants with (PR 2.50, 95% CI 1.34–4.64) and without hepatitis B infection (PR 1.73, 95% CI 1.09–2.73) (table 3). However, among participants who were not pesticide users, the increase in the risk of abnormal liver tests did not reach statistical significance in those with hepatitis B infection (PR 1.60, 95% CI 0.69–3.69). Regarding hepatitis C infection, pesticide use was associated with abnormal liver tests in both participants with (PR 10.61, 95% CI 6.00–18.77) and without the infection (PR 2.10, 95% CI 1.06–4.15). An increase in the risk of abnormal liver tests was also observed in participants with hepatitis C infection who were not pesticide users (PR 7.92, 95% CI 4.30–14.59).

**Table 3:** Risk of abnormal liver tests stratified by viral hepatitis status and pesticide use. [CI=confidence interval; HBsAg=hepatitis B surface antigen; Anti-HCV Ab=anti-hepatitis C virus antibody; AST=aspartate aminotransferase; ALT=alanine aminotransferase].

Viral hepatitis status		Pesticide use	Abnormal liver tests^†^ N (%)	Prevalence ratio (95% CI)
HBsAg				
	Negative	Non-user	26 (15.7)	1.00 (Reference)
	Negative	User	33 (27.0)	1.73 (1.09–2.73)
	Positive	Non-user	5 (25.0)	1.60 (0.69–3.69)
	Positive	User	9 (39.1)	2.50 (1.34–4.64)
Anti-HCV Ab				
	Negative	Non-user	12 (7.7)	1.00 (Reference)
	Negative	User	19 (16.2)	2.10 (1.06–4.15)
	Positive	Non-user	19 (61.3)	7.92 (4.30–14.59)
	Positive	User	23 (82.1)	10.61 (6.00–18.77)

^†^ Abnormal liver tests defined as AST or ALT >40 U/L.

In the multivariable regression analyses, a higher risk of abnormal liver tests was observed among participants with hepatitis B infection [adjusted PR (PR_adj_) 3.21, 95% CI 1.38–7.48], hepatitis C infection (PR_adj_ 23.52, 95% CI 11.30–48.96), and those who sprayed pesticides upward (PR_adj_ 3.39, 95% CI 1.52–7.54) (table 4). The increase in the risk of abnormal liver tests associated with downward spraying of pesticides did not reach statistical significance (PR_adj_ 1.52, 95% CI 0.69–3.32).

**Table 4:** Independent risk factors for abnormal liver tests from multivariable log-binomial regression analysis. [SE=standard error; PR=prevalence ratio; CI=confidence interval; HBsAg=hepatitis B surface antigen; Anti-HCV Ab=anti-hepatitis C virus antibody].

		Estimate (β)	SE	Adjusted PR ^†^	95% CI
Pesticide spraying pattern					
	Downward spraying ^‡^	0.418	0.399	1.52	0.69–3.32
	Upward spraying ^§^	1.220	0.408	3.39	1.52–7.54
Viral hepatitis status					
	HBsAg	1.166	0.432	3.21	1.38–7.48
	Anti-HCV Ab	3.158	0.374	23.52	11.30–48.96

^†^ Adjusted for age, sex, alcohol consumption, smoking status, pesticide spraying pattern, and viral hepatitis status. ^‡^ Downward spraying: pesticide application with spray gun directed toward the ground. ^§^ Upward spraying: pesticide application with spray gun directed toward the tree canopy.

### Further analyses

As a sensitivity analysis, the calculated E-value for upward pesticide spraying was 6.24, while that of hepatitis B infection was 5.87. Under the assumption of causality, the estimated PAF suggested that 30.5% of abnormal liver test cases in this population might be attributable to upward pesticide spraying.

## Discussion

This study demonstrated that pesticide exposure was associated with a higher risk of abnormal liver tests. Under the assumption of causality, the estimated PAF suggested that 30.5% of abnormal liver test cases in this population might be attributable to upward pesticide spraying, which is similar to, if not larger than, the proportion of cases attributable to hepatitis B infection.

Exposure to pesticides can occur in various agricultural activities, including mixing, loading, spraying, and equipment cleaning, as well as environmental exposure (4). Agricultural workers often experience repeated exposures to multiple pesticides, sometimes in combination with other occupational hazards (23). Despite extensive efforts to collect exposure data, accurately quantifying pesticide exposure among agricultural workers remains challenging (24). Variability in pesticide formulations, application frequency, and recall limitations often hinder the acquisition of detailed and reliable exposure information (25, 26). Therefore, we adopted a comparative approach to evaluate the effects of pesticide exposure—we compared two groups of individuals who shared similar environmental and lifestyle characteristics but differed in pesticide use and spraying patterns. The two agricultural regions may also differ in pesticide composition. In Taiwan, mango cultivation commonly involves fungicide and insecticide applications, whereas water caltrop cultivation in aquatic environments may involve a different pesticide profile (27). Because pesticide classes vary in toxicological properties, differences in pesticide mixtures across cultivation systems may contribute to exposure heterogeneity. However, as individual-level data on specific pesticide types were not available, the impact of compositional differences could not be directly evaluated and warrants further investigation.

A common mechanism of pesticide-induced toxicity involves oxidative stress damage (9). Pesticide exposure can disrupt oxidative homeostasis by increasing production of highly reactive species or impairing cellular antioxidant defenses, leading to destruction of membrane lipids, proteins, nucleic acids, and other cellular components (9, 28). Oxidative stress can affect various cellular pathways, including those involved in inflammatory responses and mitochondrial function, resulting in the disturbance of cellular and tissue homeostasis (9, 11). When such mechanisms occur concurrently or sequentially, they can promote disease development in multiple organ systems, including the brain, kidney, and liver (29). Previous studies have reported inconsistent associations between pesticide exposure and liver injury. Several investigations observed elevations in serum AST and ALT among exposed individuals (13–15, 30), while others revealed no significant differences or even lower liver enzyme levels (16, 31, 32). Most previous studies did not focus on the relationship between pesticide use and liver injury, and therefore lacked comprehensive adjustment for major confounders such as viral hepatitis, alcohol consumption, and environmental or lifestyle factors. By accounting for these factors, our study provides stronger evidence supporting the hypothesis of pesticide-induced liver injury.

To detect early hepatic effects, we used biochemical markers of liver injury rather than the prevalence of liver disease or liver-related mortality. Serum AST and ALT are sensitive indicators of hepatocellular injury, reflecting membrane permeability and cellular necrosis (19, 33). Even modest elevations of these enzymes have been associated with increased risks of liver-related morbidity and mortality (20, 34). Although aminotransferase testing alone is not sufficient to confirm pesticide-induced hepatotoxicity at the individual level, a correlation may be identified by comparing the distribution of aminotransferase abnormalities between the populations with different exposure patterns.

Abnormal liver tests are greatly influenced by viral hepatitis and alcohol consumption (20), which are potential confounders in studying hepatotoxicity. Viral hepatitis is an infectious disease that causes liver inflammation and injury. Hepatitis B and C are the leading causes of chronic hepatitis worldwide and major contributors to liver cirrhosis and hepatocellular carcinoma (35). Although pesticide exposure and viral hepatitis are not directly related, occupational exposure to hepatotoxic chemicals and hepatitis virus infection may coexist among workers (36, 37). Furthermore, pesticide exposure may act as an additive or synergistic risk factor for hepatocellular carcinoma in individuals with concurrent hepatitis B or C infection (38, 39), and thus individuals with both pesticide exposure and viral hepatitis may be at higher risk of liver injury and disease progression. Our study controlled for these potential confounding effects through stratified analysis and multivariable regression. The results demonstrated that the association between pesticide exposure and abnormal liver tests persisted among participants regardless of hepatitis infection status, suggesting that pesticide exposure is an independent risk factor for liver injury. Given these risks, individuals with both pesticide exposure and viral hepatitis infection warrant enhanced assessment and monitoring.

Our investigation found that participants who sprayed pesticides upward had a higher risk of abnormal liver tests than those who sprayed downward, likely due to differences in exposure intensity. Levels of occupational exposure during pesticide application depend on multiple factors, including application method and equipment, application duration, type of farm and crop, pesticide formulation, use of personal protective equipment (PPE), and environmental conditions (40). Crop type and height largely determine the spraying direction adopted by farmers. As crop height increases, pesticides are more likely to pass through the applicator’s breathing zone, and dermal exposure correspondingly rises when handheld sprayers are employed (41, 42). A previous study of 120 applicators categorized crops by height: <80 cm (downward spraying), 80–130 cm (horizontal spraying), and >130 cm (upward spraying). The estimated dermal exposure increased from downward to upward spraying directions, with the highest exposure for spraying upward (43). Similar findings were reported in another study involving even taller crops (44). These studies demonstrate that spraying direction substantially influences exposure levels. The Guantian District is located in the tropical area and thus has year-round hot and humid conditions, which have been reported to reduce consistent use of protective clothing among agricultural workers (45). These environmental factors may contribute to variability in PPE compliance and consequently affect exposure intensity. Accordingly, appropriate protective measures remain important to minimize applicator exposure (46).

Compared with previous studies, our study has several strengths. First, we used a random sample from the list of household registries, ensuring sample representativeness. Second, the participation rate was relatively high (82.8%), which reduces the possibility of selection bias. Third, participants had similar environmental and lifestyle characteristics, as both agricultural regions were within the same Guantian District (70.8 km^2^). This reduced the potential confounding effects of environmental and lifestyle factors. Still, several limitations should be considered. First, the cross-sectional design limits our ability to establish temporal relationships. Although associations are biologically plausible, prospective studies are needed to confirm causality. The PAF estimates assume causality and should be interpreted as hypothetical estimates. Second, residual confounding remains possible. Although the calculated E-values indicate that an unmeasured confounder would need to be strongly associated with both exposure and outcome (E-value up to 6.24) to fully explain away the observed associations, E-values do not substitute for detailed exposure characterization. Residual confounding related to unmeasured exposure dimensions cannot be entirely excluded. In addition, although smoking status was adjusted for in the analysis, quantitative measures of cumulative smoking exposure (eg, pack-years) were unavailable because the standardized questionnaire provided by the government in conjunction with the health examination did not collect information in greater detail. Third, while this study relied on self-reported exposure data, we used objective laboratory tests for outcomes, minimizing misclassification. Participants had no perceivable reason to misreport exposure based on liver test results. Therefore, the misclassifications were unlikely to bias the study results. Fourth, detailed information on pesticide types, application concentrations, cumulative exposure duration, and use of PPE was not available. Pesticide exposure is inherently heterogeneous, varying according to pesticide class, formulation, intensity, and duration of use; such variability may influence toxicological profiles and exposure levels. Moreover, cumulative working years were not measured, and we were unable to determine the proportion of participants who were actively engaged in pesticide application at the time of examination. Therefore, the observed associations should be interpreted as reflecting differences in exposure patterns rather than quantified cumulative dose or substance-specific effects. Furthermore, the two agricultural regions differ in their crop cycles, and seasonal variation in pesticide application activity at the time of blood sampling may have contributed to differences in exposure intensity. Specifically, mango harvesting in Taiwan typically occurs from May to July, and pesticides are more intensively applied from January to May, after which the fruits are bagged on the tree for protection. Water caltrop is typically planted in early June, and pesticides are more intensively applied since then to about one month before harvesting, which generally occurs between October and November. Because the health examination program was implemented in summer, participants in the water caltrop region had ongoing exposure while those in the mango region did not. As we observed a higher prevalence of abnormal liver tests in the mango region, this ongoing exposure would not change our speculation of pesticide’s adverse effects on the liver. Finally, as this study was conducted in a specific region of Taiwan, the findings may not be generalizable to areas with different agricultural practices or pesticide exposure levels.

### Concluding remarks

Our study demonstrated that pesticide use was associated with liver injury, as indicated by elevated serum ALT and AST levels. Hepatitis B and C infections were also associated with abnormal liver tests and were prevalent in this agricultural population. Nonetheless, the proportion of abnormal liver test cases in this population attributable to upward pesticide spraying might be similar to, if not larger than, the proportion of cases attributable to hepatitis B infection. Therefore, regular health monitoring is crucial for pesticide users, particularly those with viral hepatitis because they have multiple important risk factors. Pesticide users should be aware of the associated hazards and implement appropriate protective measures. Specifically, upward spraying was associated with a higher risk of abnormal liver tests compared to downward spraying. Our findings enhance the association between pesticide use and liver injury and highlight the need for longitudinal research to develop effective preventive strategies.

## Data Availability

The datasets used and analyzed during the current study are available from the corresponding author upon reasonable request.
